# Use of multivariate analysis as a tool in the morphological characterization of the main indigenous bovine ecotypes in northeastern Algeria

**DOI:** 10.1371/journal.pone.0255153

**Published:** 2021-07-26

**Authors:** Aissam Bousbia, Sofiane Boudalia, Yassine Gueroui, Kamel Hadded, Abdelkader Bouzaoui, Dounia Kiboub, George Symeon

**Affiliations:** 1 Faculté des Sciences de la Nature et de la Vie et Sciences de la Terre et de l’Univers, Université 8 Mai 1945 Guelma, Guelma, Algérie; 2 Laboratoire de Biologie, Eau et Environnement, Université 8 Mai 1945 Guelma, Guelma, Algérie; 3 Research Institute of Animal Science, HAO-DEMETER, Paralimni Giannitsa, Greece; Institute of Evolutionary Biology, Pompeu Fabra University, SPAIN

## Abstract

Sustainability in livestock farming requires monitoring of autochthonous breeds which are well adapted to the local environment. The morphometric measurements seem to be the first approach which can provide useful information on the suitability of animal genetic resources for selection. In this work, thirteen morphometric variables were used for the phenotypic characterization of 130 adult autochthones cattle randomly selected from 30 local farms in Guelma. There were cases from four commonly accepted and traditional ecotypes: Guelmois, Cheurfa, Sétifien and Fawn. The results showed several and significant positive correlations between the different variables. Correlations were analyzed using Varimax orthogonal rotation PCA and three factors were extracted, which explain more than 75% of the total variation in the four ecotypes. Stepwise discriminant analysis showed that 6 of the 13 variables had discriminatory power to define the phenotypic profile of the ecotypes. Canonical discriminant analysis indicated that the Sétifien ecotype is separate from the other three ecotypes. Mahalanobis distances were significant between the different ecotypes except for the distance between the Guelmois and Fawn ecotypes. The cross-validation procedure assigned 91.42% of the Sétifien animals to their genetic group, while the percentages of animals assigned to the Cheurfa, Guelmois and Fawn ecotypes were 80.00%, 65.71% and 53.33% respectively. The multivariate approach has proven to be effective in differentiating the four ecotypes, with clear morphological differences from the Sétifien ecotype that may benefit from a genetic improvement program for more sustainable genetic resources preservation.

## Introduction

In order to deal with the effects of globalization, urbanization, mechanization, increase in world population, global warming and climate change, it is urgently needed to transform our agriculture and livestock farming systems to more sustainable agriculture by taking into account the environmental considerations (fight against climate change, genetic characterization and monitoring of local breeds which are well adapted to the local environment, preserving and redeploying biodiversity endangered breeds) [1, 2]. Moreover, this transformation aims also to provide a fair and stable income and good working conditions to farmers and could contribute significantly to social equity and local economies [3–5].

Algeria is rich in a wide variety of indigenous cattle breeds, which are appreciated for their adaptive traits such as tolerance to heat stress and diseases resistance [6–8]. These indigenous cattle are all similar to the brown Atlas [9].They are subdivided into several subpopulations, which are phenotypically differentiated by at least a different phaneroptic and morphological character such as the color of the coat, the head and the size of the animal:

i) the “Guelmoise” ecotype, whose coat is dark gray, commonly; ii) the Cheurfa ecotype, characterized by a light gray, almost whitish coat; iii) the “Setifiénne” characterized by a uniform blackish coat; iv) the “Fawn” (Chélifien” or “Tlemcenien”), the color of the coat of which varies between brown and beige. However, this diversity has been exposed to poorly planned crossbreeding, mainly with artificial insemination based on imported semen, which has reduced drastically the number of local breeds [5, 10, 11].

To preserve this genetic diversity, and after the adoption of the Global Plan of Action for Animal Genetic Resources [12], conservation activities have been established in several countries [13], and breed characterization seems to be the first approach to the sustainable use of animal genetic resources [14].

Morphometric measurements are used to assess the characteristics of different animal breeds, and can provide useful information on the suitability of animals for selection [15–19]. In the same way, morphometric studies are used to characterize the body conformation of different animal breeds, compare the growth of different individuals, and describe individuals or populations more effectively than conventional weighing and classification methods [20, 21].

Multivariate discriminant analyses of morphological traits have been reported, in several previous morphometric studies, to be effective for a precise and objective discrimination of different population of cattle [22–28], goat [29–34], sheep [16, 35–37], horses [38] and camel [39].

This study aims to evaluate the morphological differences among the Algerian brown Atlas cattle subpopulations, and to determine the major variables/traits for the provision of maximum separation among the four local ecotypes. These morphological characters have significant role in the programs of improvement and sustainable conservation of indigenous breeds.

## Materials and methods

### Ethical statement

This study was carried out as part of the Bovisol project (Breeding and management practices of indigenous bovine breeds: Solutions towards a sustainable future www.rias.gr/bovisol) whose protocol has been approved by the Bioethics Committee on Animal Research of 8 Mai 1945 Guelma University, Algeria. The study involved taking body measurements from cattle with the consent and in the presence of the cattle herder. There is no specific legislation for body measurements and hence no approval was necessary.

### Data and sampling

**Fig 1:**
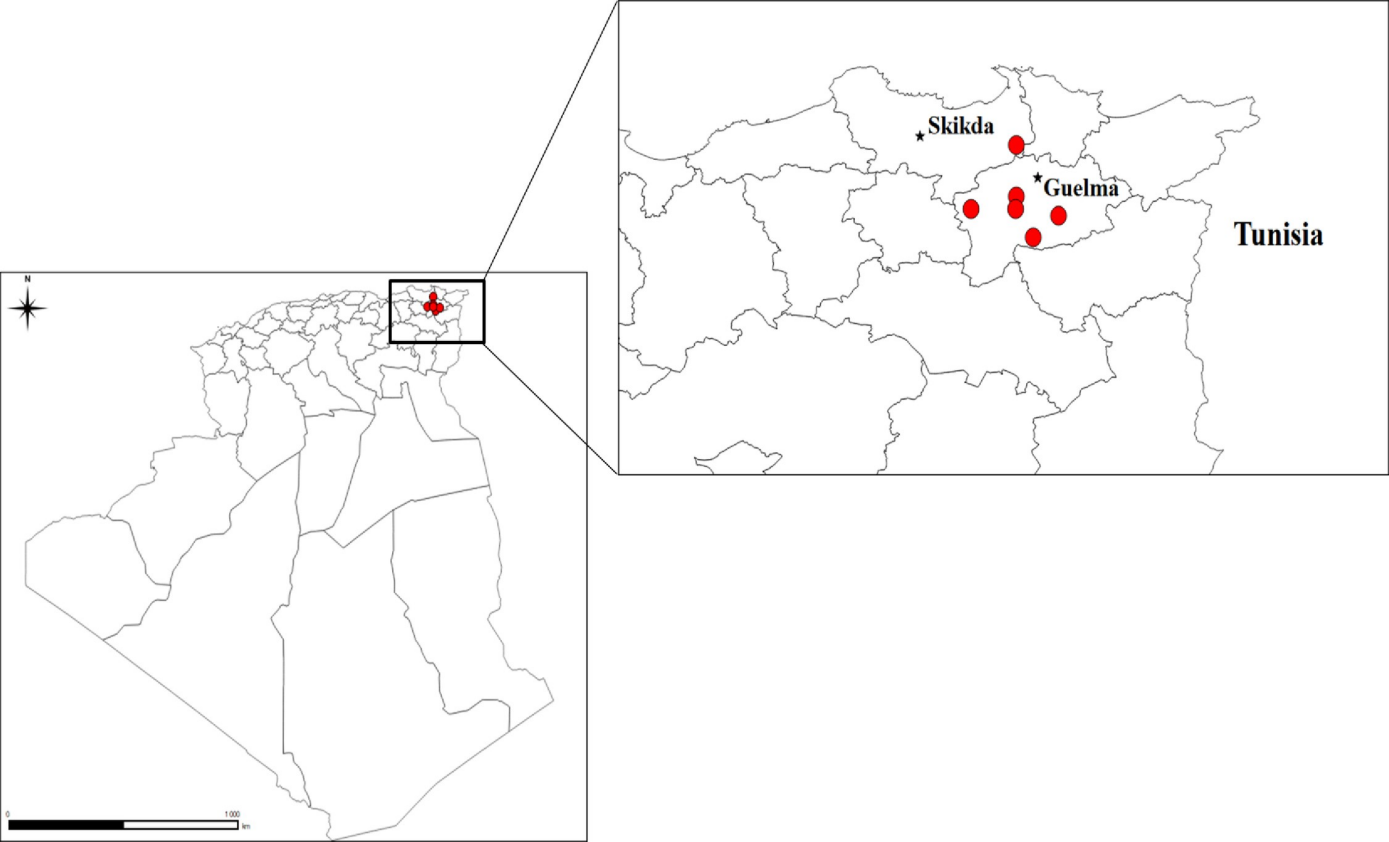
Study area. Map of Guelma (Algeria) showing the locations of the municipalities investigated. Map created using the Free and Open Source QGIS.

**Fig 2:**
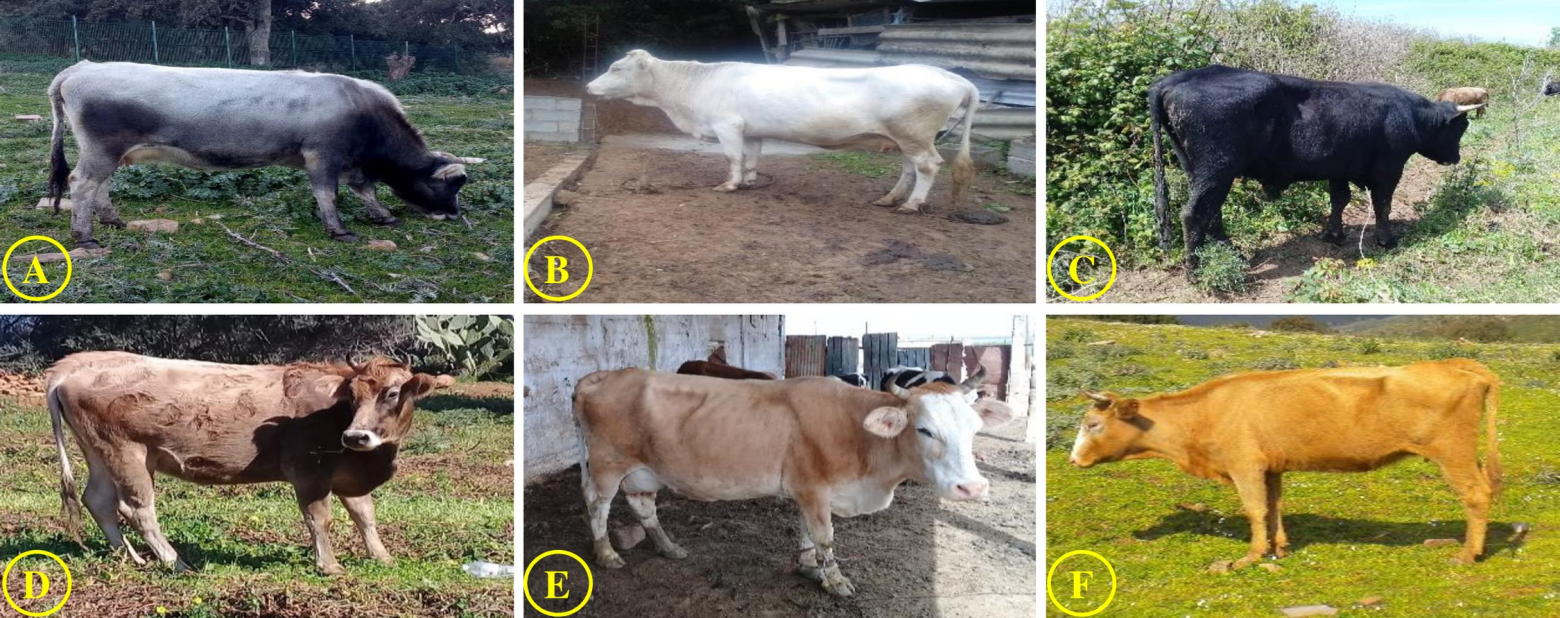
The four brown Atlas cattle ecotypes examined in the present study. (A) ecotype Guelmois; (B) ecotype Cheurfa (C); ecotype Sétifien; (D), (E) and (F) ecotype Fawn.

The present study was conducted in the northern-east of Algeria (Fig 1) from June 2018 to May 2019. The measurements came from 130 adult local (30 males and 100 females) randomly selected from 30 local farms (Table 1), and aged two years or older. They were cases from four commonly accepted and traditional ecotypes with specific characters (color of the coat, the head and the size of the animal): Guelmois (35: 25 females and 10 males), Cheurfa (30: 25 females and 5 males), Sétifien (35: 25 females and 10 males) and Fawn (30: 25 females and 5 males) (Fig 2). Local farms included in this study are characterized, on the one hand, by small herds that do not exceed ten animals in 30% of the farms, and on the other hand, the practice of extensive breeding, with low use of feed supplements.

**Table 1:** General characteristic of the study locations and number of cattle herds surveyed in Guelma region (Algeria).

Farms surveyed (no.)	Latitude N	Longitude E	Altitude (m)	Topography	Bioclimatic zones
3	36° 24′ 11″	7° 02′ 44″	533	Piedmont & Mountain	Sub-humid
13	36°15′57″	7°23′50″	892	Piedmont & Mountain	Sub-humid
4	36°22′15″	7°32′28″	420	Piedmont & Mountain	Sub-humid
1	36°42′33″	7°18′09″	101	Plain	Humid
7	36°27′46″	7°26′81″	328	Plain	Humid
2	36°24′07″	7°17′42″	422	Piedmont	Sub-humid

### Studied variables

Thirteen body measurements were assessed following the FAO guidelines for phenotypic characterization of animal genetic resources (Table 2) [40]. Animals’ age was determined according to the number of permanent incisors 2, 4, 6 and 8 and for the oldest subjects based on the declaration of the breeders. The body measurements were carried out using a measuring stick, a measuring tape or wooden caliper when the animals were standing on a level surface and maintained in upright posture by their respective owners. All the measurements were recorded by the same operator to avoid effects between operators. All traits were recorded from the left side of each animal placed on flat ground, and held by two assistants.

**Table 2:** Morphological traits measured on 130 indigenous cattle in Guelma region (Algeria).

Quantitative variable	Description
Chest girth (CG)	Circumference of the body immediately behind the scapula in a vertical plane, perpendicular to the longitudinal axis of the body.
Body length (BL)	Horizontal distance from the point of shoulder point to buttock tip (ischial tuberosity).
Height at withers (HW)	Vertical distance from the bottom of the front foot to the highest point of the shoulder on the withers.
Muzzle Circumference (MC)	Measurement must be a little above the nostrils and around the point where the dewlap meets the chin.
Muzzle width (MW)	Width of the muzzle at the level of the nostrils
Hock circumference (HC)	Measurement must be oblique, passing through the tip and the fold of the hock.
Pelvic width (PW)	Horizontal distance between the extreme lateral points of the hook bone (tuber coxae) of the pelvis.
Pelvic length (PL)	Distance between the tip of the hip and the tip of the buttocks.
Ear length (EL)	Distance between fixing point and tip of the ear.
Horn length (HOL)	Length of the horn on its outer side, from its root to the tip.
Head length (HL)	Distance between the nape at the muzzle incision.
Canon circumference (CC)	Measurement made at the level of the right barrel of the animal of the front limb.
Dactyl thoracic index (DTI)	Canon circumference/ Chest girth

### Statistical analyses

Collected data were processed and analyzed using XLSTAT Version 2016.02, Addinsoft, Paris, France (www.xlstat.com) and the IBM SPSS Statistics for Windows, Version 20 (IBM SPSS Statistics for Windows, IBM Corporation, Armonk, NY).

The descriptive statistics (mean, standard deviation and coefficient of variation) were calculated for morphometric variables. Data were analyzed with the Weighted least squares general linear model (WLS-GLM) which uses the least squares method, which included ecotype group, sex (male, female) and age group as fixed effects.

The statistical model used took the form:

$$Y_{ijk}=ecotype_{i}+sex_{(i)j}+age_{(j)K}+\varepsilon ijk.$$

Y_ijk_: is the value of the different morphometric descriptors (CG; BL; HW; MC; HC; LP; WP, EL; HOL; HL, MW, CC and DTI).

Ecotype_i_ shows the fixed effect of the different ecotypes with (i = 1 to 4; *i*1 = Guelmois ecotype, *i*2 = Cheurfa ecotype, *i*3 = Sétifien ecotype, and *i*4 = Fawn ecotype).

Sex (i) j is the fixed effect of male and female sex (j = 1 for the male, n1 = 30 and j = 2 for the female, n2 = 100).

The fixed effect of animals’ age (j) K with [K = 1 to 5; ≤ 3 (14 animals), 3 to 6 (71 animals),> 6 to 8 (28 animals), > 8 to 10 (7 animals) and > 10 years (10 animals)].

Animals were reared on farms with almost the same farming and grazing practices. Consequently, the effects of husbandry and localities were confounded with the ecotype and were not included in the model.

Interactions between effects were analyzed and significant effects were reported. For each body measurement and age, comparisons of the least squares means were carried out using the Bonferonni test after examining the effect of significance on the observed variables.

Pearson correlation coefficients were estimated for different body measurements. The Cronbach’s alpha coefficient (α) was calculated to assess the homogeneity and internal consistency of a set of variables for a given group.

In order to reduce dimensionality of our data set, principal component analysis (PCA) was performed. According to Everitt et al. [41], PCA is a method of reducing a set of multivariate data variables, X1, X2,. . ., XP, to new variables, y1, y2.. . ., yp (axis or factor), which are not correlated with each other and explain decreasing proportions of the total variance of the initial variables defined as follows:

$$y_{1}=a_{11}\;x_{1}+a_{12}\;x_{2}+\ldots \ldots +a_{1p}x_{p}$$

$$y_{2}=a_{21}\;x_{1}+a_{22}\;x_{2}+\ldots \ldots +a_{2p}x_{p}$$

$$y_{p}=a_{p1}\;x_{1}+a_{p2}\;x_{2}+\ldots \ldots +a_{pp}x_{p}$$

The coefficients being chosen so that y1, y2. . . .., yp take into account decreasing proportions of the total variance of the original variables, x1, x2,. . . ., xp. In order to test the validity of the factor analysis of the data set, the Kaiser-Meyer-Olkin (KMO) sampling adequacy criterion was calculated. The low values of KMO indicate that the correlations between any variable and the other variables are unique, and are not related to the remaining variables outside of every single correlation. Pundir et al. [20] reported that variables with a KMO of less than 0.50 should be eliminated before the factor analysis. Bartlett’s sphericity test was calculated to establish the validity of the dataset at a significance level of 1% [42]. Kaiser’s rule was used to determine the number of factors retained whose eigen value is greater than 1 [43]. The Orthogonal Rotation Method (Varimax) was used to minimize the number of variables with strong correlations, especially with the first main component. The common variance between each variable and all the components selected was calculated. The commonality for each variable measures the variance of this variable explained jointly by the set of factorial components retained [43].

Stepwise discriminant analysis was carried out to identify the morphological traits having a discriminating power between the studied ecotypes. The importance of these morphological features was assessed according to their partial R^2^ value (*P* <0.05). Linear combinations of body measurements were performed by canonical analysis, which used to perform multivariate analysis that calculated the Mahalanobis distance (D^2^) between the different cattle ecotypes.

The phenotypic discrimination among breed populations was assessed with a cross-validation test. This required the removal of 1 individual from the first matrix, and then a discriminant analysis was performed with the remaining observations to classify the omitted individual. Performance was evaluated according to the percentage of correctly and incorrectly classified bovine. The morphological distinctness of each ecotype was defined using the percentage of correctly-classified individuals.

## Results and discussion

### Comparison of measured morphometric traits among different ecotypes

A total of 130 animals which raise two different ecotypes (73%), or one ecotype (27%) were used. Differences between the different ecotypes for morphometric variables are presented in S1 Table. The body measurements (CG, BL, HW, PW and PL) showed similar means for the Guelmois and Fawn ecotypes. These results suggest that the Guelmois and Fawn ecotypes have small stature compared to the Cheurfa and Sétifien ecotypes. The later ecotypes have better skeletal and muscular development, which indicates that they are better for meat production.

For the Sétifien ecotype, the average of the chest measurement is equal 175.88 cm which is the largest in comparison with the other ecotypes. These results indicate good conformation and harmonious development of the internal organs. The body measurements observed in Sétifien and Cheurfa cattle are related to the geographic location where they are generally found in the plain while the Guelmois and Fawn ecotypes are often found in mountainous areas. This observation was confirmed by the dactyl-thoracic index which evolves in the opposite direction to the animal walking ability.

The coefficients of variation for the different measures showed a generally low variability (i.e. 70% of the variables studied recorded a coefficient of variation which does not exceed 10% which indicates that the cattle from each ecotype have similar size). These coefficients vary from 3.98% (PL) to 23.15% (HOL) for the Guelmois ecotype, from 3.05% (CG) to 15.10% (HOL) for the Cheurfa ecotype, from 3.38% (PL) to 19.82% (HOL) for the Sétifien ecotype and from 3.25% (PL) to 19.46% (HOL) for the fawn ecotype.

The length of the horns (HOL) represents the most heterogeneous biometric parameter in the ecotypes studied, which is in concordance with results reported by Boujenane [24], who showed that the homogeneity of body measurements observed from the local cattle breeds (Oulmes-Zaer and Tidili) in Morocco might be due to natural selection, favoring particular shape and size that is well adapted to the local harsh environment, which characterized by under scarce feed and fodder. Moreover, this adaptation ability was also observed in Mursi indigenous cattle breed in Ethiopia, which presents a very large morphological variability that can help the breed to survive and produce in its natural environment [44].

The analysis of variance obtained by GLM indicated that “ecotype” has a significant effect (*P* <0.05) for parameters with economic importance such as CG, HW, HC, PW and PL. These parameters are very sought in genetic improvement programs. However, no significant effect was recorded for parameters which are not important in selection programs, such as HOL, EL, CC and DTI (S1 and S2 Tables). The differences between ecotypes are in favor of “Sétifien” cattle comparing with other ecotypes for CG, HW, MC, HC, PW, PL, HL and MW.

Also, in our experimental conditions, results of all measurements show a significant effect of sexual dimorphism in the four ecotypes, which is in agreement with the data reported by Aguirre-Riofrio et al. [23] for Creole Cattle in Southern of Ecuador. However, these results should be interpreted with caution given the small sample size in male groups.

Except for a few variables studied such as HW, MC and CC “age” has no significant effect on the four ecotypes. This can be explained by the fact that the all the measurements were taken on adult animals.

A total of 78 correlations by ecotype between the different body measurements were calculated, 52 were significant, and 42 of them were positive for the Guelmois ecotype. For the Fawn ecotype, only 3 correlations were negative out of a total of 52 significant combinations. The number of significant combinations for the Sétifien ecotype was 57 with 52 positive correlations. The lowest number of significant combinations was recorded in the Cheurfa ecotype with 44 significant correlations.

As a result, most of the correlations between the variables measured varied from 36.6% to 92.6%, from 33.5% to 92.3, from 36.4% to 96.9% and from 36.7% to 94% for the Guelmois, Sétifien, Cheurfa and Fawn ecotypes respectively.

For the Guelmois ecotype, ear length (EL) was not correlated with BL, HW, MC, HC, and PW, indicating the independence of these variables with EL. This independence was more observed in the Sétifien ecotype. Also, other correlations but not significant were recorded between the length of the horns (HOL) and the other measured variables except for the Muzzle Circumference (MC) which was negatively correlated with the length of the horns (HOL) for the Cheurfa and Fawn ecotypes.

**Fig 3:**
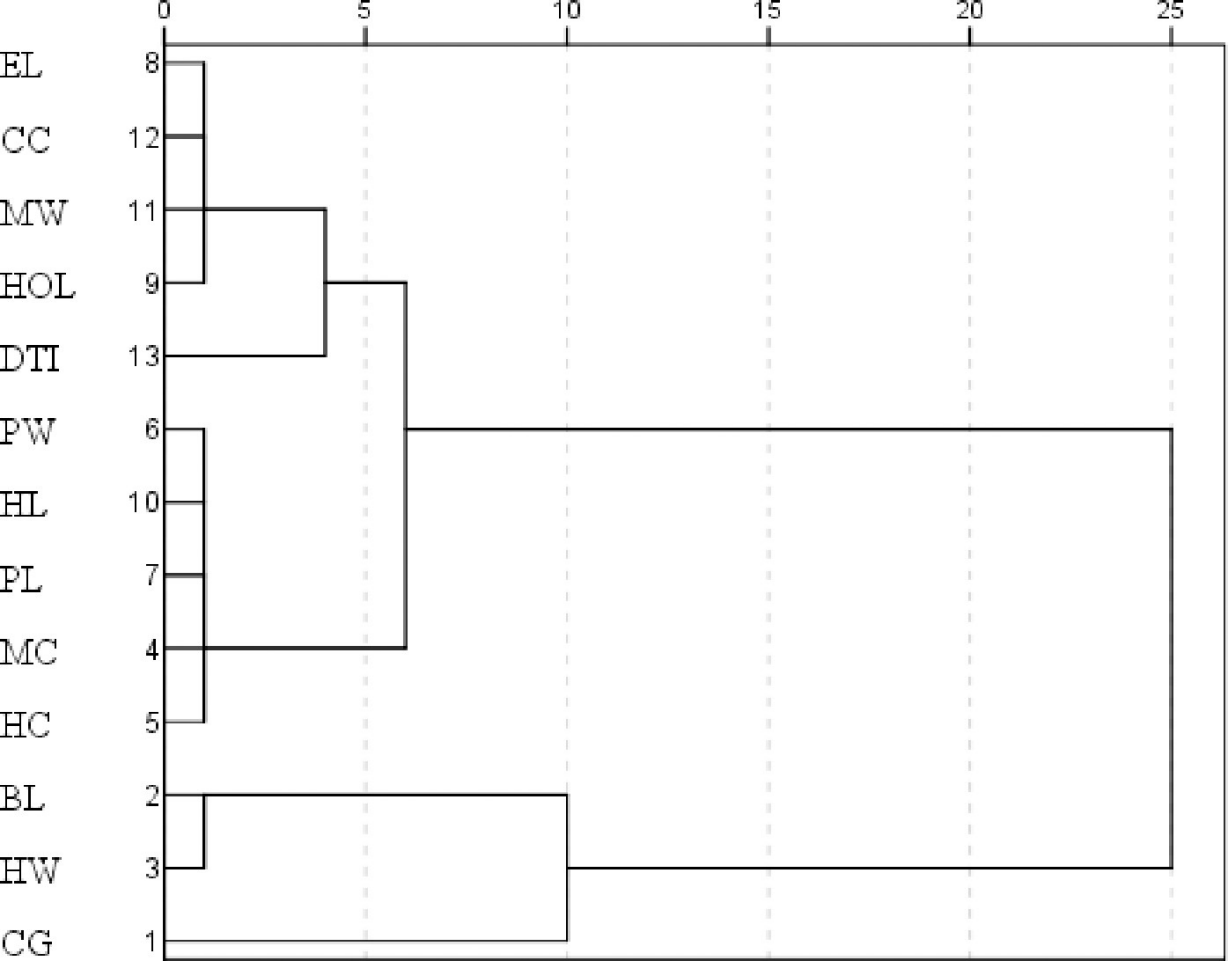
Dendrogram based on Euclidean distance. Dendrogram illustrating the morphometric relationships examined in the present study.

The correlations found in this study are stronger than those obtained in other studies across the world [15, 20, 24]. Analysis of the relationships between the 13 morphometric variables performed shows the existence of two distinct groups (Fig 3). The first group was formed by variables which define the size of the animal (CG, HW and BL), which are significantly correlated, and the second group was formed by all the others variables.

### Factorial analysis in principal components

The results obtained allow us to note an internal consistency of (0.816), (0.816), (0.875) and (0.864) for the Guelmois, Cheurfa, Sétifien and Fawn ecotypes respectively, which indicates better internal coherence, especially of the Sétifien sample. Homogeneity is satisfactory when the value of the coefficient is at least equal to 0.80 [45].

The estimate of sampling adequacy using Kaiser-Meyer-Olkin (KMO) gives an overall mean for all the variables equal to (0.622), (0.621), (0.677) and (0.680) respectively for Guelmois, Cheurfa, Sétifien and Fawn cattle.

The overall significance of the correlations tested with Bartlett’s test of Sphericity for the biometric traits was significant (*P* <0.0001) which allows a reduction in the dimension of all thirteen morphometric variables.

In this study, for Guelmois cattle, factor 1 represents 46.53% of the total variance, while the sum of the first three factors (1, 2 and 3) represents 75.79% of the total variance. For Cheurfa cattle, factors 1, 2 and 3 represent respectively 47.56%, 15.78% and 11.92%, and 75.27% of the total variance. Also, for the Sétifien and Cheurfa cattle, the first 3 factorial axes explained a cumulative amount of information of 77.48 and 76.47%, respectively (S3 Table).

Tolenkhomba et al. [14] extracted seven factors representing 64.31% of the total variance in local cows from Manipur in India. Pundir et al. [20] found three factors explaining 66.02% of the total variation from eighteen biometric traits of Kankrej cows, as well as Yakubu et al. [28] retained two factors which represent 85.37% of the total variation in White Flauni cattle.

S3 Table shows factor loadings for each principal component of biometry traits. The first factor assigned negative coefficients for MC, HC, EL and HOL for the Guelmois ecotype. The morphometric characters strongly correlated with the first factor (r > 0.7) are MW, CC, DTI and HOL. The second factor attributes positive coefficients to all the morphometric characteristics except for the ear length (EL), and this factor characterizes CG, BL, HW, MC and HC. The third factor provides information about PL; it characterizes the Guelmois ecotype according to the length of the body (BL).

For the other ecotypes, the coefficients show that the highest relative contributions to factors 1, 2 and 3 were HW, PL and MC for the Cheurfa ecotype, CG, DTI and EL, for the Sétifien ecotype and BL, PL, and HOL for the Fawn ecotype. The variables most closely associated with factor 1, regardless of the ecotype studied, were CG, BL, and HW. These variables tend to describe the general size of the animal, while the factors 2 and 3 indicate the shape of the animal, which is in concordance with the literature data [28, 46].

Also, the commonality of the variables, which represents the proportion of the variance of each of the 13 variables shared by the factor axes selected, vary from 0.619 (HC) to 0.882 (EL), from 0.449 (HC) to 0.918 (BL), from 0.630 (HOL) to 0.933 (CC) and from 0.519 (HW) to 0.918 (MC) for the Guelmois, Cheurfa, Sétifien and Fawn ecotype respectively (S3 Table).

The lower commonality of certain traits, such as the circumference of hock (HC) and the length of head (HL) in the Guelmois ecotype, the circumference of hock (HC) and the length of the horns (HOL) in the Cheurfa ecotype, the width of muzzle (MW) and the length of the horns (HOL) in the Sétifien ecotype, the height at the withers (HW), the width of muzzle (MW) and the length of the horns (HOL) in the Fawn ecotype indicate that these traits are less effective, to explain the total variation in body conformation compared to the other traits. Similar results have been reported by Tolenkhomba et al. [14]. The three factors obtained can be used to select animals based on a group of variables rather than on isolated traits. This is in agreement with the conclusions of Altarriba et al. [47] who predicted the effect of genetic improvement programs using reduced data on morphological traits which are sensitive to correlated responses to selection.

### Stepwise discriminant analysis

The stepwise discriminant analysis showed that 6 of the 13 body measurements have potential discriminatory power. Their partial R^2^ varied from 0.261 to 0.095. Then, the other traits (BL, MW, HC, EL, HOL, CC and DTI) were removed from the final model. These traits were the PL, followed by MC, CG, HL, PW and HW in decreasing order of discriminating power (Table 3). In addition, these traits are easy to monitor and can be used to assign the four ecotypes studied in separate populations, thereby reducing selection errors in future genetic improvement programs [27]. Boujenane [24] indicated that the discriminant step-by-step analysis shows that the cannon turn was the most discriminating variable between Oulmes ‐ Zaer and Tidili cattle, their partial R^2^ was 0.6. While the rump width was the most discriminating variable between Bunaji and Sokoto Gudali cattle with a partial R^2^ of 0.5824 [16].

**Table 3:** Summary of stepwise selection of traits.

Step	Variables	Partial R²	F	Pr > F	Wilks’ lambda	Pr < Lambda
1	PL	0.261	11.727	< 0.0001	0.749	< 0.0001
2	MC	0.259	11.064	< 0.0001	0.555	< 0.0001
3	CG	0.229	9.282	< 0.0001	0.428	< 0.0001
4	HL	0.125	4.423	0.006	0.375	< 0.0001
5	PW	0.141	5.023	0.003	0.322	< 0.0001
6	HW	0.095	3.177	0.028	0.291	< 0.0001

### Canonical discriminant analysis

Canonical discriminant analysis was used to obtain the function of all body measurements necessary for the separation of the four ecotypes. The analysis identified two significant canonical axes (p <0.0001) CAN1 and CAN2 representing respectively 53.55 and 45.46% of the total variation, these axes indicate a great reduction in the sampling space, with little loss (1%) to explain the total change. The third canonical axis (CAN3) was not taken into account because its values were very low (Table 4).

**Table 4:** Total canonical structure.

Variables	CAN 1	CAN 2	CAN 3
CG	0.406	**0.555**	0.221
BL	0.539	-0.035	0.189
HW	**0.611**	0.128	0.103
MC	**0.564**	-0.200	-0.215
HC	0.465	-0.151	-0.103
PW	0.327	**0.398**	0.009
PL	**0.687**	0.249	-0.030
EL	-0.050	-0.001	0.324
HOL	0.155	-0.185	0.150
HL	0.311	**-0.507**	0.018
MW	0.412	0.199	-0.340
CC	0.220	0.021	0.134
DTI	-0.048	-0.320	-0.022
Eugen values	0.907	0.770	0.017
TAV	0.535	0.990	1.00

TAV: total accumulated variance, most important variables (weight) within the CAN1 and CAN 2 given in bold

Several studies show a reduction in the sampling space with a very small loss of the total variation by conserving the first two canonical vectors [24, 27, 48]. The different tests used in multivariate analysis, namely Wilks’ lambda λ, Pillai’s trace, Lawley-Hotelling trace and Roy’s test showed a significant difference between the four ecotypes (*P* <0.0001).

The values of the first and second canonical axis enabled us to see that certain variables are correlated with the first canonical axis. Indeed, BL, HW, MC and PL are positively correlated (Table 4). This first axis represents the linear combination of the six morphological traits (CAN1 = - 0.059 CG + 0.087 HW + 0.226 MC + 0.087 PW + 0.844 PL + 0.037 HL—51.235) which could separate the Sétifien ecotype from other ecotypes. For axis 2, the CG is positively correlated, while HL is negatively correlated (CAN2 = 0.157 CG -0.098 HW—0.138 MC + 0.192 PW + 0.198 PL—0.328 HL -10.489). The second axis made it possible to separate the Guelmois and the Fawn ecotype from the other two ecotypes.

**Fig 4:**
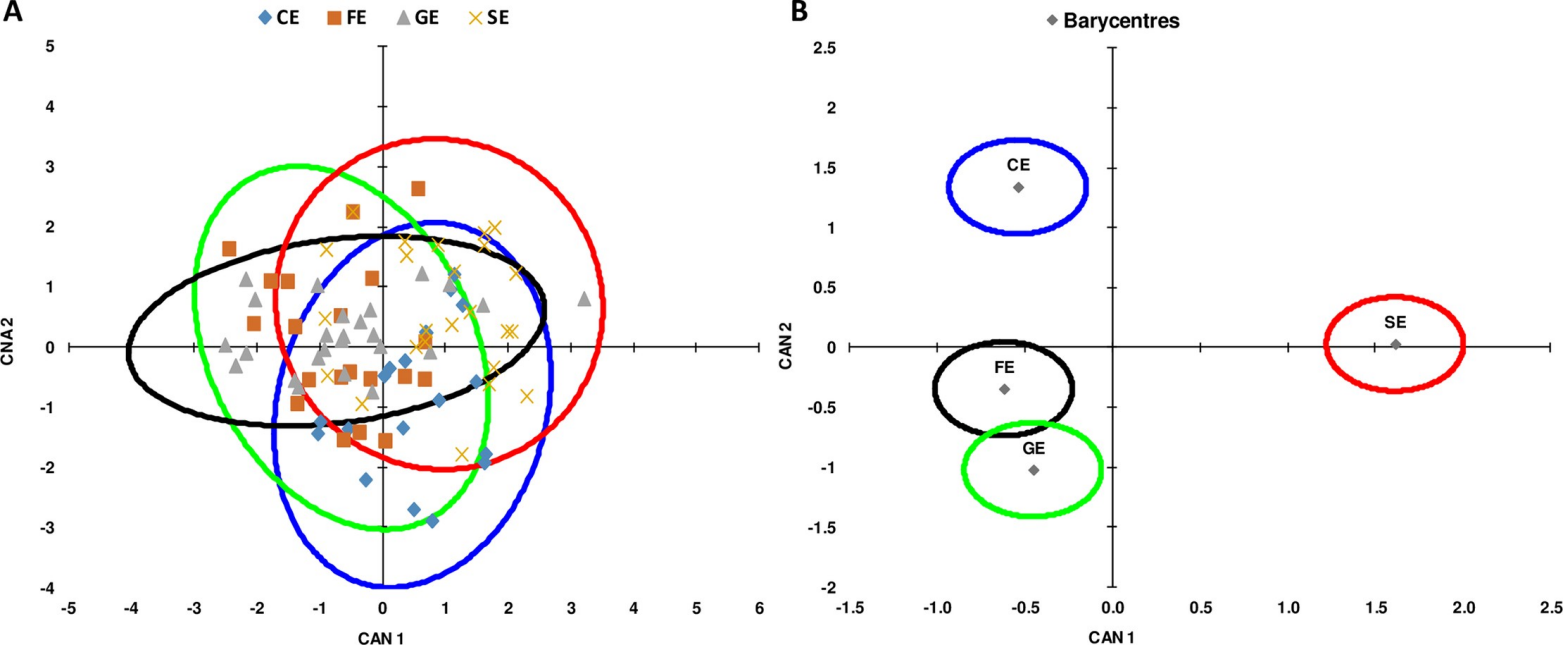
Canonical representation of the morphometric traits. (A) Association with individuals by ecotype. Canonical representation of the barycenter of four ecotypes (B).

Evaluations of cattle within each ecotype and their interrelations with other ecotypes have been shown in the projection of the four ecotypes in the plane defined by the first and the second canonical axes (Fig 4A). This projection shows a significant overlap between individuals of the Guelmois and Fawn ecotypes, which probably due to the geographic and topography zone where they are generally found in mountainous areas. The projection of the barycentres of the different ecotypes in the plane from the canonical axes illustrates two very distinct phenotypic groupings which correspond to the Sétifien and Cheurfa ecotypes. According to the first canonical axis, the Sétifien ecotype was clearly separated from the other ecotypes (Fig 4B). This was supported by the Mahalanobis distance values estimated between the four ecotypes. These distances are estimated across all characters; their comparison was carried out using the F test. The highest value was recorded between the Sétifien and Cheurfa ecotypes, while the Guelmois and Fawn ecotypes were the lowest.

From per pairs comparison, significant distance were recorded between all ecotypes (*P* <0.001) except for the one between the Fawn and Guelmois ecotypes (Table 5).

**Table 5:** Mahalanobis distance between different ecotypes.

	CE	FE	GE	SE
CE	0			
FE	2.912***	0		
GE	5.585***	0.602	0	
SE	6.357***	5.159***	5.395***	0

*** significance difference *P* < 0.001

**Fig 5:**
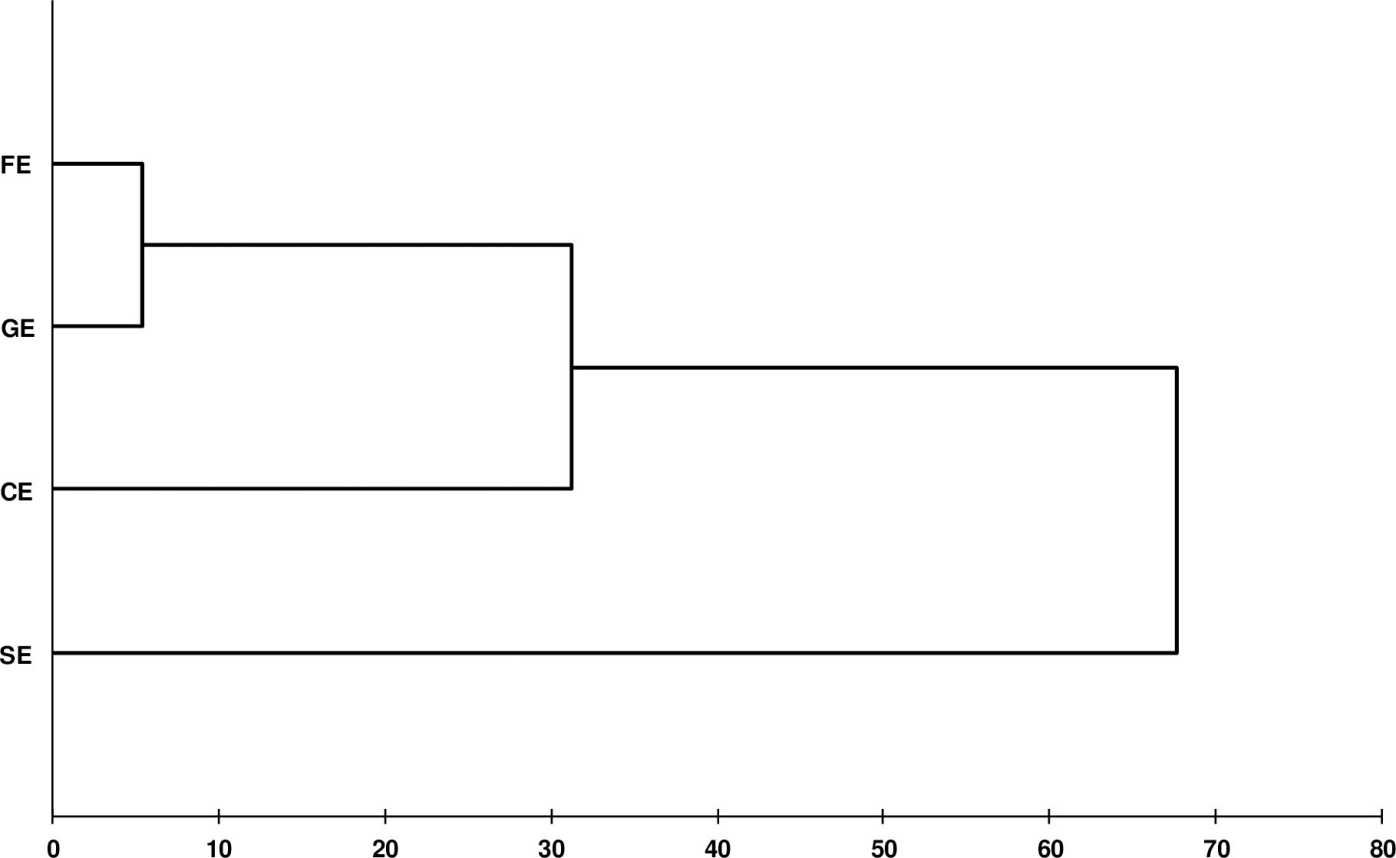
UPGMA similarity dendrogram. Euclidean distances showing clustering relationships of four ecotypes based on morphological characters.

In order to examine the relationships between the ecotypes, a phylogenetic tree was constructed from morphometric data according to the UPGMA method (Fig 5). The phylogenetic tree showed two distinct clusters, the first one contains the Guelmois, Fawn and Cheurfa ecotype, while the second cluster contains the Sétifien ecotype which is clearly separated from the other ecotypes. The representation of the similarity between the four ecotypes studied is in agreement with the results of the Mahalanobis distance (Table 5).

Using the cross-validation option in assigning each individual to their original ecotype, we show a very significant reclassification error rate (46.67%) in Fawn ecotype, followed by Guelmois, Cheurfa, and Sétifien ecotypes with 34.3%, 20% and 18% respectively (Table 6). Indeed, in the Fawn ecotype, only 53.33% of individuals have been classified in their original population, this is explained by the great heterogeneity of the Fawn ecotype which has a strong connection with the other ecotypes and in particularly the Guelmois ecotype. In addition, the Fawn ecotype cattle are localized in dispersed geographical zones which often encourage anarchic crossings with the other ecotypes. The error in reclassifying the animals is due to the significant genetic mixing between the different ecotypes.

**Table 6:** Percentages of individual classification per state based on discriminant analysis.

Ecotypes	CE	FA	GE	SE	Total	% correct
CE	24	4	0	2	30	80.00%
FE	6	16	8	0	30	53.33%
GE	1	9	23	2	35	65.71%
SE	2	0	1	32	35	91.42%
Total	33	29	32	36	130	72.61%

% of cross-validated grouped cases were correctly classified

The allocation results are in perfect agreement with results obtained from the molecular characterization of four Algerian cattle breeds (Guelmois, Cheurfa, Tlemcenienne and Zebu) using 22 microsatellite markers [49]. The authors have shown poor genetic differentiation in the cattle populations studied with F_ST_ mean value of (0.039) and high heterozygosity value (0.84). Indeed, the total genetic variation is dominated by differences among individuals (97.10%) while 2.90% among populations.

Currently, several autochthones bovine ecotypes are of mixed origin, which makes it difficult to determine the phenotype of the original breed [10]. Most individuals of the Sétifien ecotype were classified according to their original population (91.42%), indicating a reduction in the genetic flow between this ecotype and the other ecotypes studied. This situation offers a morphometric characteristic specific to the Sétifien ecotype; therefore, most of the individuals of this ecotype cannot be wrongly classified in the other ecotypes. The proportion of correctly reassigned individuals is considered to be a factor in the morphological distinction of the population [32]. Indeed, the morphological differences observed between the Sétifien ecotype and the other ecotypes studied may support the hypothesis that a large part of the morphological variation is under genetic control, indicating that they can be the subject of a genetic improvement program.

## Conclusion

In summary, the principal component analysis provided a mean of reducing the number of morphometric traits to be recorded into groups of variables that can be used to explain body conformation for animal selection program. Therefore, it is necessary to exploit specific traits of each ecotype to establish programs for sustainable genetic improvement. The characters HW, CG, MC, BL, PL and HL were the most discriminating variables to separate the four ecotypes (Guelmois, Cheurfa, Sétifien and Fawn). The study showed that the Sétifien ecotype was larger than the other ecotypes for the majority of morphometric traits. The approach used in this study could be very interesting in the assessment, management and conservation of different autochthones bovine populations, where the primary goal is to obtain local genetic resources with a pure phenotype for future selection, which is beneficial for breeding strategies improvement. Further studies, especially in molecular genetic analysis is necessary for the validation of current results.

## Supporting information

S1 Table Arithmetic means and least squares means for the different morphometric descriptors (cm) of the different ecotypes. (DOC)

S2 Table Results of the WLS-GLM for the morphological characters measured on the different ecotypes. (DOC)

S3 Table Factor pattern and communality of the body measurements with factors 1, 2 and 3 in the different ecotypes studied. (DOC)
