# Supplementary material for: Use of multivariate analysis as a tool in the morphological characterization of the main indigenous bovine ecotypes in northeastern Algeria
Source: PLoS One. 2021 Jul 26;16(7):e0255153. doi: 10.1371/journal.pone.0255153 (PMC8312925; doi:10.1371/journal.pone.0255153)
Supplement: S1 Table — (DOC) [file pone.0255153.s001.doc]

**S1 Table.** Arithmetic means and least squares means for the different morphometric descriptors (cm) of the different ecotypes

| Variables | Arithmetic means (CV) | | | | Least-squares means ± SE | | | | SL |
| --- | --- | --- | --- | --- | --- | --- | --- | --- | --- |
|  | GE, n =35 | CE, n=30 | SE, n=35 | FE, n=30 | GE, n =35 | CE, n=30 | SE, n=35 | FE, n=30 |  |
| CG | 168.57 (5.27) | 172.70 (3.05) | 175.88 (6.08) | 168.23 (4.24) | 171.08 ± 1.22 | 176.34 ± 1.01 | 179.16 ± 1.55 | 173.13 ± 1.31 | *** |
| BL | 122.85 (9.05) | 123.56 (5.22) | 128.40 (6.94) | 121.70 (6.41) | 125.42 ± 1.55 | 126.54 ± 1.11 | 129.55 ± 1.43 | 124.79 ± 1.62 | NS |
| HW | 124.34 (5.64) | 124.93 (3.69) | 128.40 (4.15) | 124.20 (3.52) | 125.18 ± 1.18 | 126.27 ± 0.94 | 129.80 ± 0.95 | 125.56 ± 0.95 | *** |
| MC | 46.14 (6.95) | 45.20 (5.35) | 48.65 (8.16) | 46.03 (7.40) | 47.03 ± 0.61 | 47.23 ± 0.47 | 50.31 ± 0.72 | 46.97 ± 0.56 | ** |
| HC | 37.60 (8.13) | 36.46 (8.31) | 39.71 (9.51) | 37.06 (3.65) | 38.73 ± 0.63 | 38.29 ± 0.06 | 40.86 ± 0.64 | 38.13 ± 0.63 | *** |
| PW | 42.00 (11.04) | 43.80 (9.65) | 45.37 (10.80) | 42.73 (11.37) | 43.62 ± 0.84 | 46.33 ± 1.03 | 46.77 ± 0.85 | 44.53 ± 0.97 | *** |
| PL | 41.14 (3.98) | 41.26 (3.17) | 42.54 (3.38) | 40.86 (3.25) | 41.61 ± 0.23 | 42.09 ± 0.23 | 42.94 ± 0.19 | 41.54 ± 0.20 | *** |
| EL | 17.60 (11.98) | 17.70 (8.75) | 18.05 (7.81) | 18.20 (8.57) | 17.23 ± 0.37 | 17.32 ± 0.35 | 18.06 ± 0.35 | 17.98 ± 0.34 | NS |
| HOL | 20.51 (23.15) | 19.33 (15.10) | 21.08 (19.82) | 20.70 (19.46) | 19.56 ± 0.78 | 18.68 ± 0.76 | 20.23 ± 0.64 | 20.40 ± 0.74 | NS |
| HL | 44.00 (7.25) | 41.56 (7.79) | 44.82 (11.55) | 43.40 (10.13) | 45.89 ± 0.59 | 43.92 ± 0.64 | 45.92 ± 0.72 | 45.25 ± 0.69 | ** |
| MW | 11.85 (12.23) | 11.93 (10.20) | 12.68 (9.06) | 11.56 (10.03) | 12.36 ± 0.16 | 12.74 ± 0.20 | 13.37 ± 0.16 | 12.45 ± 0.19 | *** |
| CC | 16.31 (13.54) | 15.96 (1.86) | 16.94 (15.05) | 15.73 (11.88) | 17.30 ± 0.16 | 17.60 ± 0.20 | 17.94 ± 0.22 | 17.32 ± 0.18 | NS |
| DTI | 0.90 (8.72) | 0.95 (8.28) | 0.96 (10.46) | 0.92 (8.30) | 0.99 ± 0.01 | 1.00 ± 0.01 | 1.01 ± 0.01 | 0.99 ± 0.01 | NS |

n: sample size per ecotype for both male and female animals, GE: ecotype Guelmois, CE: ecotype Cheurfa, SE: ecotype Sétifien, FE: ecotype Fawn, CV: coefficient of variation (%), SE: standard error, *** (*P* < 0.001). ** (*P* ≤ 0.01), SL: Significance level, NS: not significant.
