# Supplementary material for: Use of multivariate analysis as a tool in the morphological characterization of the main indigenous bovine ecotypes in northeastern Algeria
Source: PLoS One. 2021 Jul 26;16(7):e0255153. doi: 10.1371/journal.pone.0255153 (PMC8312925; doi:10.1371/journal.pone.0255153)
Supplement: S2 Table — (DOC) [file pone.0255153.s002.doc]

**S2 Table. Results of the WLS-GLM** for the morphological characters measured on the different ecotypes

| Sources | Variables | df | M.S. | F | *P* |
| --- | --- | --- | --- | --- | --- |
| Ecotypes | CG | 3 | 16.248 | 9.288 | 0.000 |
|  | BL | 3 | 4.489 | 2.578 | 0.057 |
|  | HW | 3 | 12.833 | 8.370 | 0.000 |
|  | MC | 3 | 9.251 | 6.282 | 0.01 |
|  | HC | 3 | 10.488 | 6.396 | 0.000 |
|  | PW | 3 | 11.183 | 7.884 | 0.000 |
|  | PL | 3 | 20.273 | 13.256 | 0.000 |
|  | EL | 3 | 3.735 | 2.398 | 0.071 |
|  | HOL | 3 | 2.643 | 1.551 | 0.205 |
|  | HL | 3 | 11.051 | 5.605 | 0.01 |
|  | MW | 3 | 14.487 | 9.883 | 0.000 |
|  | CC | 3 | 3.075 | 2.092 | 0.105 |
|  | DTI | 3 | 0.647 | 0.287 | 0.834 |
| Sex | CG | 1 | 45.859 | 26.216 | 0.000 |
|  | BL | 1 | 31.483 | 18.081 | 0.000 |
|  | HW | 1 | 6.029 | 3.932 | 0.050 |
|  | MC | 1 | 6.334 | 4.301 | 0.04 |
|  | HC | 1 | 13.935 | 8.498 | 0.004 |
|  | PW | 1 | 11.435 | 8.062 | 0.005 |
|  | PL | 1 | 76.131 | 49.782 | 0.000 |
|  | EL | 1 | 15.250 | 9.982 | 0.02 |
|  | HOL | 1 | 51.266 | 30.085 | 0.000 |
|  | HL | 1 | 43.455 | 21.206 | 0.000 |
|  | MW | 1 | 110.689 | 75.507 | 0.000 |
|  | CC | 1 | 396.03 | 269.360 | 0.000 |
|  | DTI | 1 | 1037.774 | 460.651 | 0.000 |
| Age | CG | 4 | 0.417 | 0.239 | 0.916 |
|  | BL | 4 | 2.648 | 1.521 | 0.2 |
|  | HW | 4 | 4.456 | 2.906 | 0.025 |
|  | MC | 4 | 28.100 | 19.081 | 0.00 |
|  | HC | 4 | 2.129 | 1.298 | 0.275 |
|  | PW | 4 | 1.252 | 0.882 | 0.477 |
|  | PL | 4 | 1.201 | 0.785 | 0.537 |
|  | EL | 4 | 0.842 | 0.541 | 0.706 |
|  | HOL | 4 | 1.902 | 1.116 | 0.352 |
|  | HL | 4 | 1.051 | 0.513 | 0.726 |
|  | MW | 4 | 1.561 | 1.065 | 0.377 |
|  | CC | 4 | 3.939 | 2.679 | 0.035 |
|  | DTI | 4 | 2.009 | 0.892 | 0.471 |

M.S: Medium square, significance difference *P* < 0.05.
