# Supplementary material for: Use of multivariate analysis as a tool in the morphological characterization of the main indigenous bovine ecotypes in northeastern Algeria
Source: PLoS One. 2021 Jul 26;16(7):e0255153. doi: 10.1371/journal.pone.0255153 (PMC8312925; doi:10.1371/journal.pone.0255153)
Supplement: S3 Table — (DOC) [file pone.0255153.s003.doc]

**S3 Table. Factor pattern and communality of the body measurements with factors 1, 2 and 3 in the different ecotypes studied**

|  | GE | | | | CE | | | | SE | | | | FE | | | |
| --- | --- | --- | --- | --- | --- | --- | --- | --- | --- | --- | --- | --- | --- | --- | --- | --- |
| Traits | PC1 | PC2 | PC3 | C | PC1 | PC2 | PC3 | C | PC1 | PC2 | PC3 | C | PC1 | PC2 | PC3 | C |
| CG | 0.371 | 0.727 | 0.434 | 0.855 | 0.853 | 0.176 | -0.100 | 0.769 | 0.880 | 0.264 | 0.001 | 0.844 | 0.593 | 0.369 | 0.499 | 0.737 |
| BL | 0.368 | 0.793 | 0.101 | 0.774 | 0.881 | 0.255 | 0.278 | 0.918 | 0.551 | 0.433 | -0.484 | 0.726 | 0.920 | -0.064 | -0.007 | 0.850 |
| HW | 0.216 | 0.841 | 0.051 | 0.756 | 0.938 | 0.025 | 0.003 | 0.880 | 0.865 | 0.114 | 0.044 | 0.764 | 0.455 | 0.255 | 0.497 | 0.519 |
| MC | -0.456 | 0.742 | -0.098 | 0.768 | 0.277 | -0.028 | 0.747 | 0.635 | 0.770 | -0.341 | 0.270 | 0.782 | 0.907 | -0.123 | 0.282 | 0.918 |
| HC | -0.002 | 0.771 | 0.155 | 0.619 | 0.414 | 0.223 | 0.477 | 0.449 | 0.815 | 0.277 | 0.317 | 0.842 | 0.834 | 0.283 | 0.074 | 0.781 |
| PW | 0.352 | 0.681 | -0.417 | 0.761 | 0.713 | -0.598 | -0.054 | 0.869 | 0.725 | 0.360 | -0.121 | 0.671 | 0.507 | 0.474 | 0.436 | 0.672 |
| PL | 0.630 | 0.389 | 0.348 | 0.669 | 0.178 | 0.889 | 0.003 | 0.822 | 0.394 | 0.594 | -0.399 | 0.667 | -0.129 | 0.919 | 0.127 | 0.877 |
| EL | -0.240 | -0.053 | -0.906 | 0.882 | -0.170 | -0.670 | -0.511 | 0.740 | 0.168 | -0.082 | 0.896 | 0.838 | 0.000 | -0.534 | 0.671 | 0.736 |
| HOL | -0.818 | 0.228 | 0.072 | 0.727 | -0.288 | -0.045 | 0.611 | 0.458 | 0.019 | -0.783 | 0.125 | 0.630 | -0.047 | -0.111 | -0.791 | 0.640 |
| HL | 0.632 | 0.438 | 0.268 | 0.663 | 0.837 | 0.246 | 0.086 | 0.768 | 0.772 | 0.326 | -0.331 | 0.812 | 0.621 | 0.692 | 0.154 | 0.888 |
| MW | 0.864 | 0.309 | -0.018 | 0.842 | 0.515 | 0.728 | -0.298 | 0.884 | 0.675 | 0.432 | -0.111 | 0.655 | 0.655 | 0.397 | 0.232 | 0.640 |
| CC | 0.765 | 0.346 | 0.373 | 0.844 | 0.776 | 0.407 | 0.309 | 0.863 | 0.569 | 0.780 | -0.026 | 0.933 | 0.692 | 0.653 | 0.082 | 0.912 |
| DTI | 0.789 | 0.067 | 0.259 | 0.694 | 0.634 | 0.419 | 0.391 | 0.730 | 0.257 | 0.919 | -0.017 | 0.911 | 0.592 | 0.638 | -0.127 | 0.774 |
|  |  |  |  |  |  |  |  |  |  |  |  |  |  |  |  |  |
| E | 6.049 | 2.606 | 1.199 | - | 6.183 | 2.052 | 1.550 | - | 6.774 | 2.278 | 1.020 | - | 6.615 | 50.882 | 50.882 | - |
| SV (%) | 46.531 | 20.043 | 9.224 | - | 47.564 | 15.788 | 11.927 | - | 52.111 | 17.526 | 7.843 | - | 1.960 | 15.080 | 65.962 | - |
| AV (%) | 46.531 | 66.574 | 75.798 | - | 47.565 | 63.352 | 75.279 | - | 52.111 | 69.638 | 77.481 | - | 1.367 | 10.517 | 76.479 | - |

PC: Principal component; C: communality; E: Eigen values; SV: simple variation; AV: Accumulated variation.
